# Arabidopsis WRKY50 and TGA Transcription Factors Synergistically Activate Expression of *PR1*

**DOI:** 10.3389/fpls.2018.00930

**Published:** 2018-07-13

**Authors:** Rana M. F. Hussain, Arsheed H. Sheikh, Imran Haider, Mussa Quareshy, Huub J. M. Linthorst

**Affiliations:** ^1^Institute of Biology, Leiden University, Leiden, Netherlands; ^2^School of Life Sciences, University of Warwick, Coventry, United Kingdom; ^3^Laboratory of Plant Physiology, Wageningen University, Wageningen, Netherlands

**Keywords:** AtWRKY50, TGA, Arabidopsis, *PR1*, SAR, EMSA

## Abstract

Arabidopsis *PR1* is a salicylic acid (SA) inducible marker gene for systemic acquired resistance (SAR). However, the regulation of *PR1* in plants is poorly understood. In this study, we showed that AtWRKY50 transcription factor binds to two promoter elements of *PR1* via its DNA binding domain. Interestingly, the DNA-binding sites for AtWRKY50 deviate significantly from the consensus WRKY binding W-box. The binding sites are located in close proximity to the binding sites for TGA transcription factors. Transactivation experiments in Arabidopsis protoplasts derived from wild type, *npr1-1* and *tga256* mutant plants indicated that AtWRKY50 alone was able to induce expression of a *PR1*::β-*glucuronidase* (GUS) reporter gene, independent of TGAs or NPR1. However, co-expression of TGA2 or TGA5 with AtWRKY50 synergistically enhanced expression to high levels. Yeast-2-hybrid assays and bimolecular fluorescence complementation (BiFC) experiments revealed that AtWRKY50 could interact with TGA2 and TGA5. Using electrophoretic mobility shift assays (EMSA) it was established that AtWRKY50 and TGA2 or TGA5 simultaneously bind to the *PR1* promoter. Taken together, these results support a role of AtWRKY50 in SA-induced expression of *PR1*.

**Highlights:** AtWRKY50 specifically binds to LS10 region of *PR1* promoter and interacts with TGAs to synergistically activate *PR1* expression.

## Introduction

Upon pathogen attack plants mobilize inducible defense systems. A classic example is the systemic acquired resistance (SAR) which is effective against a broad range of pathogens. The signal transduction route leading to SAR involves the induced synthesis of the endogenous signal molecule salicylic acid (SA from here onward). SAR is accompanied by the de-novo synthesis of pathogenesis-related (PR) proteins of which many directly affect pathogen growth and disease proliferation. Although their exact function is still not fully characterized, the plant kingdom-wide conserved PR1 proteins are generally considered as marker proteins for SAR. Expression of the *PR* genes is tightly regulated to fine tune plant growth and defense (Linthorst, 1991; van Verk et al., 2008).

*PR* gene expression was shown to be enhanced by SA in *Nicotiana tabacum* by the group of Katagiri et al. (1989). This augmentation in expression was dependent on the presence of so called activation sequence-1 (as-1), a DNA element in the 90 bp core promoter consisting of two TGACG tandem repeats (Qin et al., 1994). The as-1 element is specifically bound by the tobacco ASF-1 protein complex, containing basic leucine zipper (bZIP) type TGACGTCA *cis*-element-binding protein (TGA) (Katagiri et al., 1989; Qin et al., 1994; Niggeweg et al., 2000a). TGA proteins are conserved among different plant species and bind to their cognate binding sites as dimers (Boyle et al., 2009).

Also, promoters of several *PR* genes, such as *Arabidopsis thaliana PR1* and tobacco *PR-1a* contain as-1-(like) elements in promoter regions important for SA-inducible expression. In tobacco, the as-1-like element in the *PR-1a* promoter consists of a set of inverted TGACG motifs, which bind to TGA transcription factors (Strompen et al., 1998; Niggeweg et al., 2000b; Grüner et al., 2003). Likewise, a linker scanning analysis of the Arabidopsis *PR1* promoter region responsible for induced expression by the SA analog 2,6-dichloroisonicotinic acid (INA) revealed the presence of an as-1 element with two TGACG direct repeats in inverted orientation, of which one is a positive regulatory element (-645 to -636 upstream of the transcription start site; for convenience this region will further be referred as LS7, the name of the linker that was used to mutate this element), while the other (LS5, -665 to -656) mediates negative regulation of *PR1* expression (Lebel et al., 1998). Further studies revealed that additional elements other than LS5 and LS7 are also involved in INA-inducible *PR1* expression (Pape et al., 2010). In these studies, mutation of LS5 resulted in a relatively small (less than 2-fold) enhancement of the level of inducible expression in comparison with the wild type promoter, while mutation of LS7 modestly reduced expression (approximately 3-fold). Moreover, when both LS5 and LS7 were mutated, *PR1* promoter-driven expression was 2-fold higher than that of the wild type promoter, while in all these cases expression remained inducible by the SA-analog INA.

The ankyrin repeat protein NPR1 (NON-EXPRESSER OF PATHOGEN-RELATED GENE 1) plays a central role in *PR* gene induction and subsequent SAR establishment (Delaney et al., 1995; Cao et al., 1997; Wang et al., 2006). Pathogen-induced accumulation of SA induces a change of the redox state of the cell, resulting in the release of reduced NPR1 monomers from multimeric complexes residing in the cytoplasm, which subsequently translocate to the nucleus where they interact with TGA transcription factors to activate gene expression (Zhang et al., 1999; Després et al., 2000; Kinkema et al., 2000; Zhou et al., 2000; Mou et al., 2003). Through knockout analyses it was shown that Arabidopsis TGA2, TGA5, and TGA6 act as redundant but essential activators of *PR1* expression and subsequently SAR activation (Zhang et al., 2003; Kesarwani et al., 2007). Interestingly, these TGAs are constitutive repressors required for basal repression of *PR1* under non-stress conditions (Zhang et al., 2003; Rochon et al., 2006). It is believed that the interaction of the BTB/POZ domain of NPR1 neutralizes the repression domain of TGA2, while NPR1’s transactivation domain activates gene expression (Boyle et al., 2009). Recently, it was shown that coactivation by NPR1 occurs in a pulse-wise manner and is regulated by proteosomal degradation of NPR1 (Spoel et al., 2009; Fu et al., 2012).

In addition to TGAs, WRKY transcription factors are important for stress induced transcriptional reprogramming (Eulgem and Somssich, 2007; Pandey and Somssich, 2009). WRKY transcription factors are classified as a family of plant-specific DNA-binding proteins characterized by the presence of the peptide sequence Trp-Arg-Lys-Tyr (WRKY) followed by a Zn-finger domain (Rushton et al., 2010). An ever-increasing number of publications indicate the involvement of WRKY transcription factors in SAR. Unlike the TGA transcription factors that are present at steady state levels (Johnson et al., 2003), many of the WRKY genes are transcriptionally activated upon biotic and abiotic stress. Various WRKY proteins positively regulate resistance against necrotrophic pathogens, like AtWRKY33 (Zheng et al., 2006), others positively regulate defense against biotrophs, like AtWRKY53 and AtWRKY70 (Wang et al., 2006). In addition, WRKY proteins like AtWRKY18, -40 and -60 have dual effects n plant defense, either enhancing defense against biotrophic pathogens and diminishing defense against necrotrophs, or vice versa (Xu et al., 2006; Wang et al., 2006; Shen et al., 2007). Of the 74 *WRKY* genes in Arabidopsis, 49 are differentially expressed upon *Pseudomonas syringae* infection or treatment with SA (Dong et al., 2003). Most of the WRKY proteins bind to the W-box, a DNA motif with the core sequence TTGAC(T/C) and the overrepresentation of this motif in several defense related genes suggests that their expression is regulated by WRKY transcription factors (Eulgem and Somssich, 2007). Furthermore, for several WRKY genes, SA-induced expression is dependent on NPR1 and TGAs, suggesting a similar activation strategy as was originally proposed for *PR1* (Dong et al., 2003; Wang et al., 2006). Despite the fact that extensive genetic information has been obtained on the physiological processes in which specific WRKYs are involved, surprisingly little is known about which specific genes they regulate.

In the same linker scanning study that identified the as-1-like regulatory element in the Arabidopsis *PR1* promoter, a nearby consensus W-box motif (LS4, -675 to -666) with a strong negative effect was identified, suggesting that WRKY factors are important for SA-mediated *PR1* gene expression (Lebel et al., 1998). The tobacco *PR-1a* promoter does not harbor a consensus W-box, however, NtWRKY12, was found to bind to a WK-box (TTTTCCAC) that was located 13 bp proximal to the as-1-like element in the tobacco *PR-1a* promoter (van Verk et al., 2008). Mutation of the WK-box sharply reduced SA-mediated *PR-1a::GUS* expression (van Verk et al., 2008). Furthermore, pull-down assays and Fluorescence Resonance Energy Transfer (FRET) analysis showed that NtWRKY12 specifically interacted with tobacco TGA2.2 (van Verk et al., 2011). These results indicate that NtWRKY12 and TGA2.2 interact in the regulation of tobacco *PR-1a* promoter activity.

In addition to the as-1 element and the W-box, the Arabidopsis *PR1* promoter contains another nearby element that influences *PR1* expression. Mutation of this LS10 element (-615 to -606) resulted in loss of INA-inducible expression, indicating the sequence as a positive regulatory element (Lebel et al., 1998; Pape et al., 2010). Based on the presence of the sequence TTTC, LS10 has been suggested to be a potential binding site for DOF transcription factors, although experimental data to support this are lacking (Yanagisawa, 2004). Taken together, these results suggest that TGA proteins are not the only transcription factors important for *PR1* expression, but regulation of expression is mediated by additional transcription factors binding to the intricate mosaic of elements in the *PR1* promoter, and especially underline the importance of transcription factors binding to sites in LS4 and LS10.

In the present study, we identified AtWRKY50 as an activator of *PR1* gene expression and determined its binding sites in the promoter. In addition, we investigated its ability to interact with TGA2 and TGA5 and to synergistically enhance transcriptional activation.

## Results

### AtWRKY50 Is the Most Effective WRKY Activator of *PR1* Gene Expression

**FIGURE 1:**
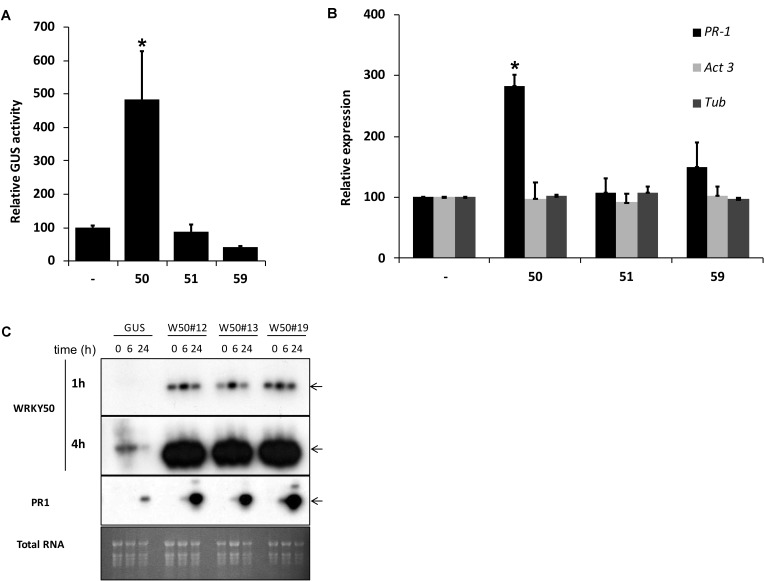
**(A)** AtWRKY50 activates the *PR1* promoter. Arabidopsis protoplasts were co-transfected with a *PR1::GUS* construct together with empty pRT101 expression plasmid (minus sign) or with plasmids containing *35S::AtWRKY50* (50), *35S::AtWRKY51* (51) or *35S::AtWRKY59* (59). After incubation, GUS activity was measured spectrophotometrically. Expression levels (%) are given relative to the expression level without WRKY effector. **(B)** Effect of AtWRKY50, AtWRKY51, and AtWRKY59 on the expression of endogenous Arabidopsis genes. Expression of *PR1*, *Act3* (actin) and *Tub* (tubulin) genes in Arabidopsis protoplasts was measured by qRT-PCR. Expression of each gene was measured in protoplasts transfected with the empty pRT101 vector (minus sign) or with the pRT101 vector containing the *35S::AtWRKY50* (50), *35S::AtWRKY51* (51), or *35S::AtWRKY59* (59) expression constructs. Bars represent the average level of mRNA accumulation observed in three experiments. mRNA levels in protoplasts transfected with the empty pRT101 vector were taken as 100%. Error bars represent the SEM. The experiment was repeated three times with similar results. Statistical differences among the samples is labeled with asterisk (*p* < 0.05). **(C)** Overexpression of *AtWRKY50* enhances SA-induced expression of *PR1* in plants. Three lines of transgenic seedlings overexpressing *AtWRKY50* (W50#12, #13, #19) and a line expressing GUS were incubated for the indicated times (in hours) in liquid medium containing 0.3 mM SA, after which RNA was extracted and analyzed for accumulation of mRNA corresponding to genes *AtWRKY50* and *PR1*. The blot hybridized with the *AtWRKY50* probe was exposed for 1 and 4 h. A similar blot was hybridized with the *PR1* probe. Arrows to the right of the blots indicate the expected positions of the respective mRNAs as deduced from the positions of the rRNAs. An Ethidium bromide stained gel showing total RNA is included as a loading control.

Previously, we identified NtWRKY12 as a transcriptional activator of tobacco *PR-1a* gene expression (van Verk et al., 2008). To investigate if WRKY transcription factors are also involved in activation of Arabidopsis *PR1* gene expression a protoplast transactivation assay (PTA) was established where 40 of the 74 Arabidopsis WRKYs proteins were screened (Wehner et al., 2011). Briefly, a fragment containing approximately 1000 bp upstream of the transcription start site of the *PR1* gene was cloned in front of the coding sequence for firefly luciferase (LUC). Parallel co-transfections of Arabidopsis protoplasts with this reporter plasmid and expression vectors containing the panel of WRKY genes under control of the 35S promoter showed that AtWRKY50 was the most effective activator of the *PR1::LUC* reporter (Supplementary Table S1). AtWRKY50 is a small, 173 amino acid long protein that belongs to a small subgroup of WRKY proteins in which the domain that interacts with the DNA is characterized by the sequence WRKYGKK as opposed to WRKYGQK present in most other WRKY proteins (Yamasaki et al., 2005). Interestingly, NtWRKY12 also belongs to this GKK subgroup (van Verk et al., 2008). In addition to AtWRKY50, only two other Arabidopsis WRKY proteins, AtWRKY51 and AtWRKY59, possess the WRKYGKK sequence and of these three, AtWRKY50 has the highest homology to tobacco NtWRKY12 (68% sequence similarity). Since constructs corresponding to AtWRKY51 and AtWRKY59 were not present in the initial screen, separate transactivation assays in Arabidopsis protoplasts were done with 35S expression plasmids for these WRKYs co-expressed with a *PR1::GUS* reporter construct. While AtWRKY50 enhanced *GUS* expression approximately 5-fold, AtWRKY51 and AtWRKY59 did not increase expression over the background level (**Figure 1A**). The overexpression of AtWRKY50 also resulted in activation of endogenous *PR1* gene expression as revealed by qRT-PCR (**Figure 1B**). In agreement with the co-expression experiment of **Figure 1A**, expression of AtWRKY51 and AtWRKY59 did not result in enhanced endogenous *PR1* mRNA accumulation. Stable AtWRKY50 transgenic overexpressor plants also showed a significantly higher *PR1* expression after 0.3 mM SA treatment than control (GUS) plants (**Figure 1C**). Taken together, we have shown that AtWRKY50 is the only Arabidopsis WRKY in the WRKYGKK group that specifically activates the *PR1* gene expression.

### AtWRKY50-Induced *PR1* Expression Is Independent of NPR1, TGA2, TGA5 or TGA6 Transcription Factors

**FIGURE 2:**
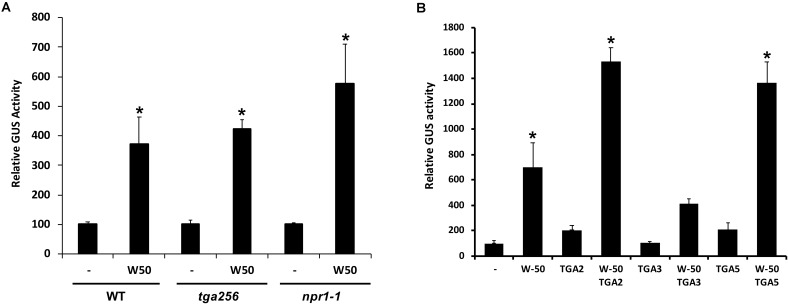
**(A)** AtWRKY50 induced expression is independent of NPR1 and TGAs. Protoplasts from wild type (WT), *tga2-1 tga5-1 tga6-1* triple mutant (*tga256*) and *npr1-1* mutant (*npr1*) plants were co-transfected with *PR1::GUS* reporter construct alone (minus sign) or together with expression plasmids containing *35S::AtWRKY50* (W50), as indicated. After incubation GUS activity was measured spectrophotometrically. The bars represent the percentage of GUS activity from triplicate experiments relative to that of the protoplasts co-transfected with the *PR1::GUS* construct and empty vector control. Error bars represent the SEM. Asterisk (^∗^) shows the statistical differences among samples (*p* < 0.05). **(B)** Synergistic effect of AtWRKY50 and TGA2 or TGA5 on *PR1* expression. Arabidopsis protoplasts were co-transfected with *PR1::GUS* reporter construct alone (minus sign) or together with expression plasmids containing *35S* promoter-controlled genes encoding AtWRKY50, TGA2, TGA3, TGA5, or combinations, as indicated. After incubation GUS activity was measured spectrophotometrically. Expression levels (%) are given relative to the expression the level without expression plasmid. The bars represent the percentage of GUS activity from triplicate experiments relative to that of the protoplasts co-transfected with the corresponding *PR1::GUS* construct and empty vector control. Error bars represent the SEM. Asterisk (^∗^) shows the statistical differences among samples (*p* < 0.05).

SA-induced expression of *PR1* genes in plants is dependent on NPR1 and it is generally assumed that NPR1 activates expression through its interaction with TGAs binding to the promoter. To investigate if the activation of the *PR1* promoter by AtWRKY50 requires NPR1 or TGAs, transactivation assays were performed with protoplasts derived from *npr1-1* mutant and *tga256* triple mutant Arabidopsis plants. AtWRKY50 was able to activate the *PR1::GUS* reporter gene in these mutant backgrounds to similar levels as in wild type protoplasts (**Figure 2A**). Apparently, *PR1* activation by AtWRKY50 did not require the TGAs or their co-activator NPR1.

Although AtWRKY50 alone is able to activate the *PR1* promoter, TGA factors may function in further modulation of gene expression. To determine the effect of TGA factors on AtWRKY50-activated *PR1* expression, Arabidopsis protoplasts were co-transfected with the *PR1::GUS* reporter construct and plasmids containing 35S promoter-driven *AtWRKY50*, *TGA2*, *TGA3* and *TGA5* genes. While AtWRKY50 enhanced *GUS* expression approximately 7-fold, the TGA proteins alone could enhance expression only 2-fold (**Figure 2B**). However, combinations of AtWRKY50 and TGA2 or TGA5 boosted expression of the reporter gene up to 14-fold, while co-expression with TGA3 did not further enhance AtWRKY50-dependent *GUS* expression. The results indicate that TGA2 and TGA5 act synergistically with AtWRKY50 to maximize activation of the *PR1* promoter.

### AtWRKY50’s C-terminal Half Binds to the *PR1* Promoter

**FIGURE 3:**
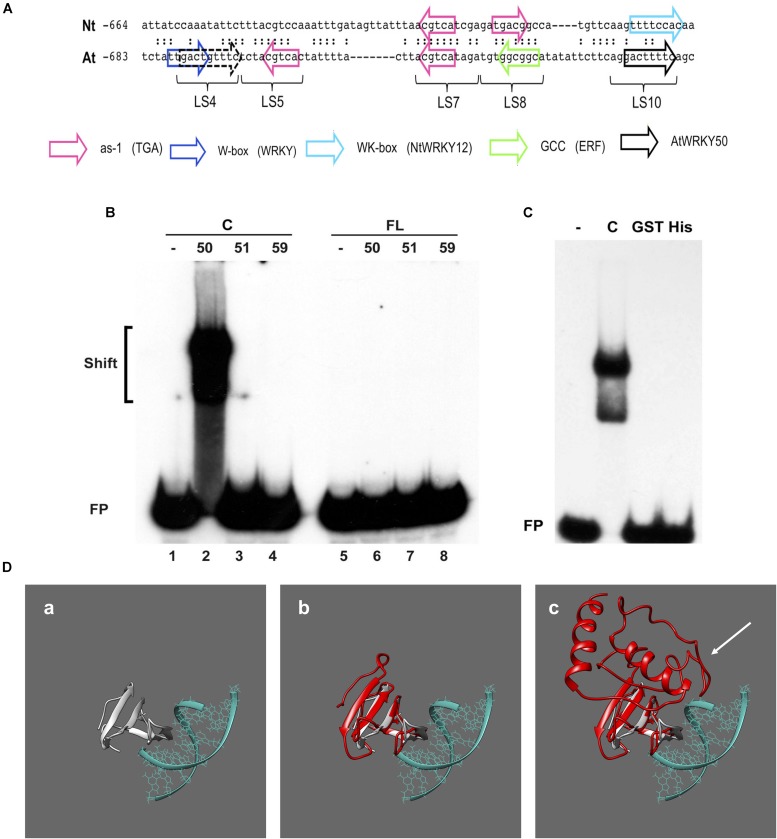
AtWRKY50 binds to the *PR1* promoter. **(A)** Comparison of sequences in the promoters of tobacco *PR1a* (Nt) and Arabidopsis *PR1* (At). Only the sequence of the top strands is given. The sequences of the promoter regions are shown with gaps to allow maximal alignment. The position of the leftmost nucleotide relative to the transcription start site is indicated. Corresponding nucleotides are indicated by colons. Colored block arrows mark consensus binding sites for various transcription factors, as indicated. The direction of the arrow indicates whether the consensus sequence is in the top (right-pointing arrow) or bottom strand. The dashed and solid black arrows mark the binding sites for AtWRKY50. The positions of sequence elements used in the linker scanning analysis of the *PR1* promoter by Lebel et al. (1998) are indicated (LS). **(B)** EMSAs were performed with an 80-bp fragment of the *PR1* promoter and GST-tagged C-terminal halves (Lanes C) or full-length (Lanes FL) versions of AtWRKY50, -51 and -59, as indicated above the lanes. **(C)** EMSAs were performed with the same probe together with the GST-tagged C-terminal half (Lane C) and GST-tagged (Lane GST) and His-tagged (Lane His) full-length versions of AtWRKY50. In **(B,C)**, lanes labeled with the minus sign were loaded with the probe only. The positions of shifts and the unbound probe (FP) are indicated. **(D)** Modeling prediction analysis of C-terminal and full-length AtWRKY50 protein binding to DNA *in silico*. **(a)** shows the structure of C-terminal WRKY4-C domain bound to DNA from Yamasaki et al. (2012) onto which AtWRKY50 and its truncated form are superimposed. The overlap of the β-sheets and overall structure correlating with interactions is shown in **(b)**. The full length AtWRKY50 sequence also demonstrates a superimposable C-terminal region **(c)** but the additional protein structure was predicted to possess a large disordered region (shown with white arrow).

Previous work on the Arabidopsis *PR1* promoter has shown that the region between approximately -700 and -600 bp upstream of the transcription start site is important for inducible gene expression upon treatment with the SA analog INA (Lebel et al., 1998). In addition to two inverted TGACG motifs (CGTCA in LS5 and LS7) comprising the as-1-like element, this region contains a consensus WRKY binding W-box (in LS4) and an additional sequence stretch (LS10). Mutational analyses revealed that all these elements are involved in INA-inducible expression. The schematic representation of the Arabidopsis *PR1* promoter and a comparison to the tobacco *PR-1a* promoter is shown in **Figure 3A**.

To analyze if AtWRKY50, AtWRKY51, and AtWRKY59 are able to specifically bind to this region of the promoter, we set up electromobility shift assays (EMSA) with an 80-bp fragment of the *PR1* promoter, corresponding to the region of -685 to -606, which covers all of the above elements. EMSAs were performed with affinity purified glutathione-*S*-transferase (GST)-coupled fusion products of the respective WRKY proteins expressed in *Escherichia coli*. Both full-length WRKYs and C-terminal halves containing both the WRKY- and Zn-finger domains were produced (Supplementary Figure S1). The GST-tagged, 88-amino acid long C-terminal half of AtWRKY50 (AtWRKY50-C) efficiently bound to the probe (**Figure 3B**, lane 2), but surprisingly, full-length GST-tagged AtWRKY50 did not produce a shift (**Figure 3B**, Lane 6). The GST-tagged full-length AtWRKY51 and AtWRKY59 did not produce band shifts as expected (**Figure 3B**, Lanes 7 and 8) and neither did their C-terminal halves (**Figure 3B**, Lanes 3 and 4), indicating that amino acids outside of the conserved WRKYGKK domain are also important determinants for specificity of binding to the *PR1* promoter. This is similar to the observations where C-terminal of NtWRKY12 was shown to bind the tobacco *PR-1a* promoter much more efficiently than full-length NtWRKY12 (van Verk et al., 2008). It is unlikely that the relatively large GST-tag fused at the N-terminus of the full-length protein masks the WRKY’s DNA-binding domain for interaction with the DNA. However, when fused to the C-terminal half, it possibly leaves the binding domain exposed, since an EMSA with full-length AtWRKY50 fused to the much smaller His-tag also failed to produce a shift with the 80-bp promoter fragment (**Figure 3C**). In order to ascertain the absence of an interaction with the full-length protein we employed homology modeling for both full length and truncated proteins. We compared the models to a known structure for a related WRKY protein’s C-terminus with DNA bound to it (Yamasaki et al., 2012), where we observe a similar series of β-sheet features that interact with the major grove of the DNA (**Figure 3Da**). When the C-terminal AtWRKY50 model is overlaid the models are superimposable, sharing similar features and corroborating the DNA binding ability (**Figure 3Db**). This contrasts with the full-length model (**Figure 3Dc**), which although possessing similar C-terminal features, has an N-terminal region that is predicted to be bulky with some regions of disorder (white arrow, **Figure 3Dc**). From these comparisons we postulate that the N-terminal region possibly impedes the binding of DNA and could explain our observations.

### Characterization of AtWRKY50’s Binding Site

**FIGURE 4:**
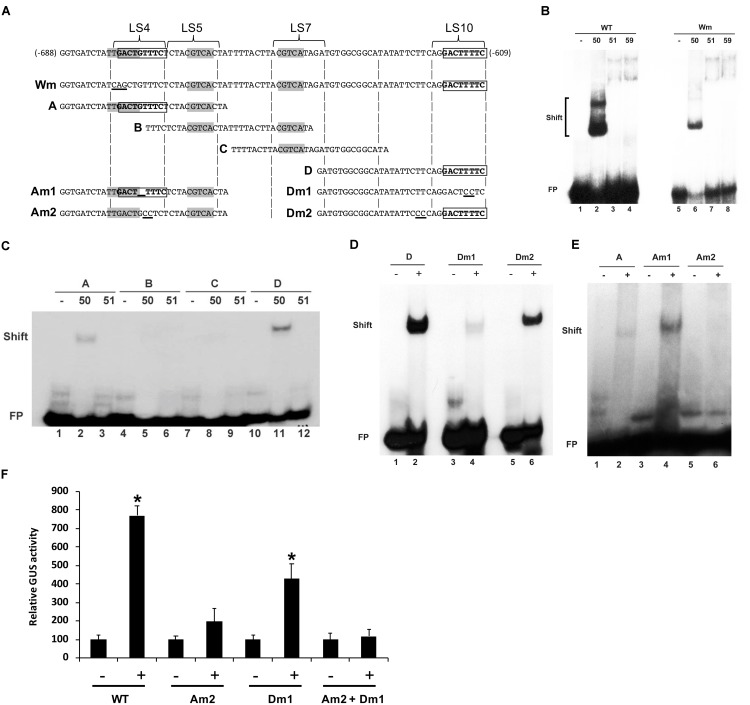
Characterization of AtWRKY50’s binding sites on the *PR1* promoter **(A)** Sequences of *PR1* promoter fragments used for EMSAs. Only the sequence of the upper strand is given. The top line displays the sequence of the 80-bp fragment corresponding to bp –688 to –609 upstream of the transcription start site. Regions LS4, LS5, LS7, and LS10, as used in the linker scanning analysis of Lebel et al. (1998), are indicated. Wm indicates an 80-bp fragment with a mutation (TTGACT to TCAGCT) in the W-box in LS4. Overlapping subfragments A, B, C, and D, and their mutant versions Am1, Am2, Dm1 and Dm2 are aligned with the sequence of the 80-bp fragment. The W-box (TTGACT) and the CGTCA boxes of the *as-1* element are indicated in bold. Mutations in Wm, Am1, Am2, Dm1, and Dm2 are underlined. **(B)** EMSAs were performed with wild type 80-bp *PR1* promoter fragment (WT) or with an 80-bp fragment with a mutation in the W-box (Wm) as probes together with the GST-tagged C-terminal halves of AtWRKY50, -51 and -59, as indicated above the lanes. The positions of band shifts and the unbound probe (FP) are indicated. Lanes labeled with the minus sign were loaded with probe only. **(C)** AtWRKY50 binds to the *PR1* promoter at two positions. EMSAs were performed with overlapping *PR1* promoter fragments A, B, C, and D as probes and GST-tagged AtWRKY50-C or the C-terminal half of AtWRKY51, as indicated above the lanes. The positions of band shifts and the unbound probe (FP) are indicated. Lanes labeled with the minus sign were loaded with probe only. **(D,E)** AtWRKY50 binds to the LS10 and LS4 element in the *PR1* promoter. EMSAs were performed with wild type **(D)** and WT **(A)** and mutant versions (Dm1, Dm2) of fragment D and Am1, Am2 of fragment A of *PR1* promoter as probes and GST-tagged AtWRKY50-C, as indicated above the lanes. The positions of band shifts and the unbound probe (FP) are indicated. Lanes labeled with the minus sign were loaded with probe only; lanes labeled with the plus sign were loaded with the probe and AtWRKY50-C. **(F)** *PR1* activation by AtWRKY50 requires intact binding sites. Arabidopsis protoplasts were co-transfected with WT and mutant *PR1::GUS* construct alone (minus sign) or together with expression plasmids *35S::AtWRKY50*. After incubation GUS activity was measured spectrophotometrically. Expression levels (%) are given relative to expression level without WRKY effector. Statistical differences among the samples is labeled with asterisk (*p* < 0.05).

To investigate if the W-box in LS4 is the binding site for AtWRKY50, a mutant version of the 80-bp fragment was constructed, in which the TTGACT sequence of the W-box was changed to TCAGCT (**Figure 4A**, probe Wm). While incubation of the wild type and mutant 80-bp probes with the C-terminal halves of AtWRKY51 and AtWRKY59 did not result in shifts (**Figure 4B**, Lanes 3, 4, 7, 8), AtWRKY50-C (C-terminal half) produced shifts with both probes (**Figure 4B**, Lanes 2, 6). Interestingly, a double shift is produced with the wild type probe, while with the mutant probe the higher shift is lost. This suggests that AtWRKY50-C has two binding sites in the 80-bp *PR1* promoter fragment of which one overlaps with the W-box in LS4. The shift with the mutant probe indicates that AtWRKY50-C also binds to a second site in the 80-bp promoter fragment, which is different from the W-box consensus.

To further delimit the AtWRKY50 binding sites in the 80-bp fragment, a series of overlapping subfragments (A to D) were generated as shown in **Figure 4A**. As expected, incubation with the AtWRKY51-C peptide did not result in shifts with any of the four subfragments (**Figure 4C**, Lanes 3, 6, 9, 12). However, AtWRKY50-C produced shifts with subfragments A and D (**Figure 4C**, Lanes 2 and 11, respectively). The shift with subfragment A supports the result from the EMSA shown in **Figure 4B**, suggesting that the sequence overlapping with the W-box in LS4 facilitates AtWRKY50-C binding. The shift with fragment D indicates the presence of an additional AtWRKY50 binding site, possibly in the LS10 element, which is different from the canonical W-box. To test this, double-stranded oligonucleotides corresponding to fragment D, containing mutations in the LS10 element (Dm1, **Figure 4A**) and upstream of the LS10 element (Dm2, **Figure 4**) were used as probes in EMSAs with AtWRKY50-C. Whereas the mutation of two nucleotides immediately upstream of the LS10 element (Dm2) did not change the ability of the probe to bind (compare **Figure 4D**, Lanes 2 and 6), mutation of two central T nucleotides in LS10 (Dm1) almost completely abolished binding of AtWRKY50-C (**Figure 4D**, Lane 4). This indicates that LS10 indeed contains a binding site for AtWRKY50, which is distinct from the consensus WRKY binding site (W-box).

Almost an exact copy of the sequence GACTTTTC of LS10 is present in LS4, partly overlapping with the W-box and with only a G inserted between the first and second T. An EMSA with the Am1 probe, in which this G was removed from subfragment A, shows that this results in enhanced binding (**Figure 4E**). We speculate that the binding of AtWRKY50-C to fragment A (**Figure 4C**, Lane 2) is actually caused by the presence of this LS10-like GACTGTTTC sequence, rather than by the W-box, as mutation of GACTGTTTC to GACTGCCTC (Am2, **Figure 4A**), which leaves the W-box intact, completely abolished binding to AtWRKY50-C (**Figure 4E**, Lane 6). The reduced binding of AtWRKY50-C observed upon mutation of the W-box (**Figure 4B**, Lane 6) could thus be caused by the fact that the W-box mutation changes the two left most nucleotides of the LS10-like element.

To test whether these promoter elements are necessary for activation of gene expression *in planta*, Am2 and Dm1 mutations (**Figure 4A**) were incorporated into the 1000 bp promoter of *PR1::GUS* reporter gene constructs, which were used in Arabidopsis protoplasts co-expression studies (**Figure 4F**). Mutation of the binding site in LS10 (Dm1) resulted in the reduction of *GUS* expression by approximately 50%, while mutation of the binding site in LS4 (Am2) reduced the expression to less than 20%. When both mutations were incorporated in the *PR1* promoter, AtWRKY50 was no longer able to activate *GUS* expression (**Figure 4F**).

Taken together, the results of these experiments support the notion that GACT(G)TTTC is the binding site of AtWRKY50 and that both these sites in the *PR1* promoter are required for maximal activation by AtWRKY50.

### AtWRKY50 Interacts With TGA2 and TGA5

**FIGURE 5:**
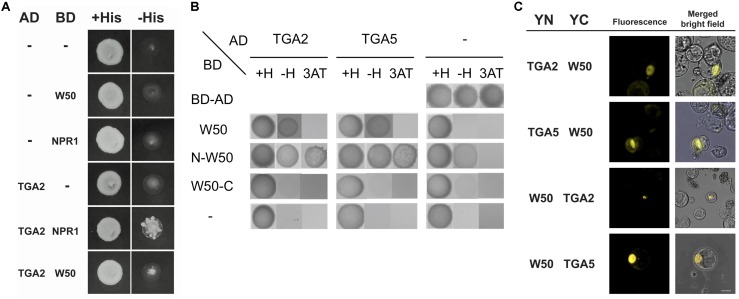
AtWRKY50 interacts with TGA2 and TGA5. **(A)** Yeast was transformed with expression plasmids pAS2.1 and pACT2, containing the coding regions of the binding domain (BD) and activation domain (AD) of GAL4, respectively. The BD domain was either fused to the coding regions of AtWRKY50 (W50) or NPR1, or was not fused (minus sign); the AD domain was not fused (minus sign) or was fused to the coding region of TGA2. Growth of transformed yeast was evaluated on medium containing histidine (+His) or minus histidine (–His). **(B)** The N-terminal of AtWRKY50 interacts with TGA2 and TGA5. Yeast was transformed with expression plasmids pAS2.1 and pACT2, containing the coding regions of the binding domain (BD) and activation domain (AD) of GAL4, respectively. The BD domain was either fused to the coding regions of full length AtWRKY50 or its N-terminal or C-terminal half. The AD domain was not fused (minus sign) or was fused to the coding region of TGA2 or TGA5. Growth of transformed yeast was evaluated on medium containing histidine (+H), minus histidine (–H) and minus histidine plus 10 mM 3AT (3AT). **(C)** AtWRKY50 interacts with TGA2 and TGA5 in Arabidopsis protoplasts. YFP fluorescence and merged bright field images of Arabidopsis protoplasts co-transformed with expression plasmids containing constructs encoding TGA2, TGA5, and AtWRKY50 (W50) fused to the N-terminus (YN) or the C-terminus (YC) of yellow fluorescent protein. Scale bar = 10 μm.

Previously, we found that the close proximity of the binding sites for NtWRKY12 and TGAs in the promoter of the tobacco *PR1a* gene may be functionally relevant for bringing both proteins together in order to direct full transcriptional activation. Further support for this came from studies that showed that NtWRKY12 interacted with TGA2.2 when expressed in yeast and in Arabidopsis protoplasts (van Verk et al., 2011). Similar to the *PR1a* promoter of tobacco, the AtWRKY50 binding sites in LS4 and LS10 and the TGA-binding as-1 element (in LS5 and LS7) of the Arabidopsis *PR1* promoter are in close proximity (**Figure 3A**). To investigate if Arabidopsis TGAs and AtWRKY50 can interact, we performed yeast-two-hybrid assays. The full-length coding sequence of AtWRKY50 was fused to the binding domain (BD) of GAL4 and co-expressed with coding sequences for TGA2 and TGA5 fused to the GAL4 transcriptional activation domain (AD) in yeast containing a *GAL1::HIS3* reporter gene. Growth of yeast was scored on media with and without histidine. TGA2 and TGA5 interacted weakly with AtWRKY50 as co-expression of *TGA2-AD* or *TGA5-AD* with *AtWRKY50-BD* could grow on a dropout medium (**Figure 5A**). The known interaction between TGA2 and NPR1 served as a positive control for this experiment. However, *HIS3* expression was relatively low, since addition of 10 mM 3-amino-1,2,4-triazole (3AT) inhibited growth. Nevertheless, this indicates that AtWRKY50 and the TGAs interacted in the yeast system. Additional two-hybrid assays with N- and C-terminal halves of AtWRKY50 indicated that the TGA interaction domain is present in the N-terminal half of the protein and that this domain provides for a strong interaction resulting in high expression of HIS3 that was not inhibited by 10 mM 3AT. The C-terminal half of AtWRKY50 clearly did not interact with the TGAs (**Figure 5B**).

The interaction of AtWRKY50 with TGAs was confirmed using bimolecular fluorescence complementation (BiFC) assays. To this end, the full-length AtWRKY50 and TGA2 or TGA5 coding sequences were fused at the N- or the C-terminus to the N- (YN) or C- (YC) terminal halves of the yellow fluorescent protein (YFP), respectively. Protein-protein interaction was analyzed 16 h after co-transfection of Arabidopsis protoplasts with expression plasmids harboring these constructs by determining the fluorescence of reconstituted YFP using confocal laser scanning microscopy. For all combinations with YN- and YC-fused versions of AtWRKY50 and TGA2 or TGA5, fluorescence was visible most strongly in nuclei, indicating a predominant nuclear presence of the AtWRKY50 and TGA fusion proteins (**Figure 5C**). Controls with combinations of unfused YN and YC or with combinations of AtWRKY50 and TGA2 or TGA5 in which only one of the proteins was fused to a YFP half did not result in fluorescence (Supplementary Figure S2). The results indicate that AtWRKY50 interacts with both TGA2 and TGA5.

### AtWRKY50-C and TGA2 or TGA5 Bind to the *PR1* Promoter Simultaneously

**FIGURE 6:**
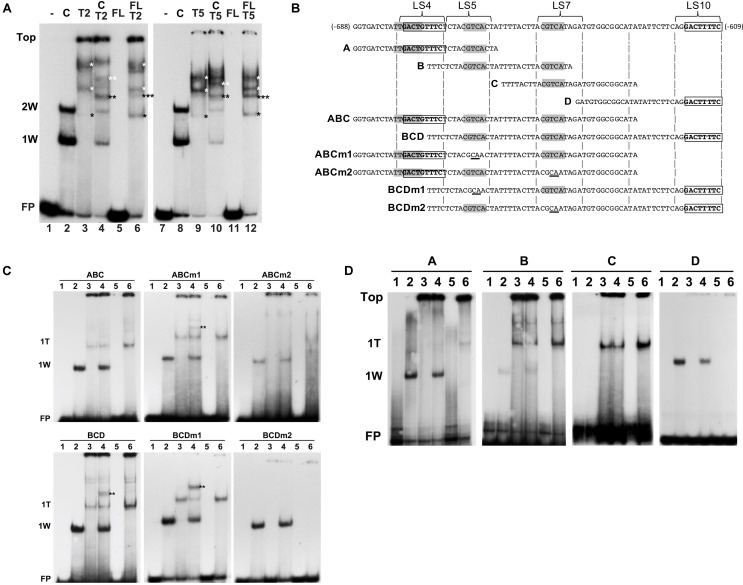
AtWRKY50 and TGA2 and TGA5 bind to the *PR1* promoter. **(A)** EMSAs were performed with an 80-bp fragment of the *PR1* promoter without protein (minus signs) or with GST-tagged C-terminal half (C) or full-length (FL) versions of AtWRKY50, and His-tagged TGA2 (T2) or TGA5 (T5), and combinations of these proteins, as indicated above the lanes. The positions of the unbound probe (FP), the top of the gel (Top), and of band shifts caused by one (1W) or two (2W) AtWRKY50-C proteins are indicated at the left. Shifts caused by binding of single (single black asterisks) or multiple (single white asterisks) TGA proteins and shifts caused by a combination of TGA and AtWRKY50 (double black asterisks) are indicated. **(B)** Sequences of *PR1* promoter fragments used for electromobility shift assays. Overlapping subfragments A, B, C, and D, and subfragments ABC and BCD are aligned with the sequence of the 80-bp fragment. The overlapping W-box (TTGACT) and AtWRKY50 binding sequence (GACTGTTTC) in LS4, the CGTCA boxes of the *as-1* element in LS5 and LS7, and the AtWRKY50 binding sequence (GACTTTTC) in LS10 are indicated in bold. Subfragments ABCm1, ABCm2, BCDm1 and BCDm2 represent variants of fragments ABC and BCD with mutations (underlined) in the CGTCA boxes in LS5 and LS7, respectively. **(C)** EMSAs were performed with probes corresponding to *PR1* promoter fragments ABC and BCD and their mutated versions ABCm1, ABCm2, BCDm1, BCDm2, as indicated above the panels. EMSA incubation mixtures contained no protein (Lanes 1), AtWRKY50-C (Lanes 2), TGA2 (Lanes 3), AtWRKY50-C and TGA2 (Lanes 4), full-length AtWRKY50 (Lanes 5), and full-length AtWRKY50 and TGA2 (Lanes 6). The positions of the unbound probe (FP) and of band shifts caused by AtWRKY50-C (1W) or TGA2 (1T) are indicated at the left. Shifts caused by binding of a combination of TGA and AtWRKY50 (double black asterisks) are indicated. **(D)** EMSAs were performed with *PR1* promoter fragments A, B, C, and D as probes, as indicated above the panels. EMSA incubation mixtures contained no protein (Lanes 1), AtWRKY50-C (Lanes 2), TGA2 (Lanes 3), AtWRKY50-C and TGA2 (Lanes 4), full-length AtWRKY50 (Lanes 5), and full-length AtWRKY50 and TGA2 (Lanes 6). The positions of the unbound probe (FP), the top of the gel (Top) and of band shifts caused by AtWRKY50-C (1W) or TGA2 (1T) are indicated at the left.

Next, we investigated how combinations of AtWRKY50 and TGA2 or TGA5 influenced the binding to DNA. Therefore, EMSAs were performed with purified, *E. coli*-expressed GST-tagged AtWRKY50-C and His-tagged TGA2 and TGA5. A double shift resulting from the binding of one and two AtWRKY50-C peptides to the 80-bp *PR1* promoter was observed (**Figure 6A**, Lanes 2 and 8). A number of band shifts were observed with the EMSAs with TGA2 and TGA5 of which the intensity increased with decreasing mobility (**Figure 6A**, Lanes 3 and 9). The presence of multiple shifts with TGA proteins (notably with TGA2 and TGA5) could be ascribed to possible different degrees of occupancy of the binding sites present in the probe (Miao et al., 1994; Miao and Lam, 1995; Pontier et al., 2001; Boyle et al., 2009). However, the possibility of aggregate formation during incubation, due to non-specific interactions of these TGAs could not be excluded. Nevertheless, we speculate that the weak bands in Lanes 3 and 9 (indicated by single black asterisks) represent complexes in which only one of the CGTCA binding sites in either LS5 or LS7 is occupied by a TGA dimer. The calculated molecular weights of complexes of the 80 bp probe with a TGA-His fusion protein dimer (125 kD) or with two GST-AtWRKY50-C fusion proteins (122 kD) are almost identical and this corresponds well with the similar mobilities of the respective bands in lanes 3/9 and 2/8. The more slowly migrating bands in Lanes 3 and 9 (indicated by white asterisks), represent higher order TGA shifts, possibly including shifts in which TGAs are bound at both the CGTCA sites in LS5 and LS7. Incubation of the 80-bp probe with combinations of AtWRKY50-C and TGA2 or TGA5 resulted in the formation of new bands indicated by the double asterisks in black and white (**Figure 6A**, Lanes 4 and 10). It is highly plausible that these new bands represent complexes of the probe with both AtWRKY50-C and the respective TGA dimers (black double asterisks, MW 161 kD) and TGA oligomers (white double asterisks).

Next, we thought to investigate which of the two AtWRKY50 binding sites allows formation of a complex between AtWRKY50 and TGA. For this, promoter fragments consisting of the regions encompassing subfragments A, B and C (**Figure 6B**, ABC) and subfragments B, C and D (**Figure 6B**, BCD) were tested in EMSAs with AtWRKY50-C and TGA2. The ABC and BCD promoter fragments each contain only one of the AtWRKY50 binding sites. The single shifts (1W) observed in Lanes 2 correspond to AtWRKY50-C binding to the sites in LS4 of probe ABC and LS10 of probe BCD, respectively (**Figure 6C**). TGA2 predominantly binds to only one binding site in ABC and BCD (1T), while the presence of weak, high shifts in Lanes 3 suggests that binding of multiple TGA2 proteins occurred at low frequency. This is in contrast to the EMSAs with the longer 80-bp probe, which indicated that the TGAs preferentially bound as multimeric complexes (**Figure 6A**, Lanes 3 and 9). Yet, the ABC and BCD probes were only 18 bp shorter than the 80-bp probe, while all contained the same two CGTCA motifs. This suggests that the size of the probe determines the efficiency and number of TGA2 proteins it can bind. Obviously, the EMSAs with TGA2 alone resulted in a single prominent shift, suggesting that only one of the two CGTCA motifs in the fragments efficiently bound TGA2. To find out which of the CGTCA motifs is bound by TGA2, fragments ABCm1, BCDm1, ABCm2 and BCDm2 (**Figure 6B**), with mutations in LS5 (m1) and LS7 (m2), respectively, were used as probes in EMSAs (**Figure 6C**, middle and right panels). Apparently, mutation of the CGTCA box in LS7 interfered with TGA2 binding to the fragments (right panels), while mutation of the CGTCA motif in LS5 had no effect on binding of TGA2 (middle panels). These results were confirmed by the EMSAs of **Figure 6D** that show that fragment A, which contains the CGTCA motif in LS5, did not produce a shift upon incubation with TGA2, whereas predominantly single TGA shifts were present with probe B, containing both CGTCA motifs of LS5 and LS7, and with probe C that contains only the TGA binding site in LS7. As expected, probes A and D bind AtWRKY50-C. These results indicate that the CGTCA box in LS7 is the main binding site of TGA2. Furthermore, the shifts indicated by the double asterisks in **Figure 6A**, Lanes 4 in the panels with probes BCD and BCDm1 show that these fragments are able to bind AtWRKY50-C and TGA2 simultaneously and with higher efficiency than probe ABCm1.

### AtWRKY50 Stimulates Binding of TGA2 and TGA5 to the *PR1* Promoter

As was already shown, AtWRKY50-C binds highly efficiently and specifically to the DNA while full length AtWRKY50 was unable to bind the *PR1* promoter. We speculated that a conformational change is required to release the N-terminal half of AtWRKY50 from blocking the C-terminal *PR1* promoter binding domain. To investigate if such a release could be brought about by the interaction with TGA2 or TGA5, EMSAs were performed with the 80-bp probe and mixtures of full-length AtWRKY50 and TGA2 or TGA5. The results are shown in **Figure 6A**, Lanes 6 and 12. In addition to bands corresponding to TGA dimers and oligomers (black and white single asterisks, respectively; compare to Lanes 3 and 9), surprisingly, the EMSAs resulted in an extra band shift (indicated by the triple asterisks) that migrated to a similar position as the band shifts in Lanes 4 and 10, respectively, that correspond to the probe binding combinations of WRKY50-C and TGA (double asterisks). It is conceivable that these triple asterisks band shifts in Lanes 6 and 12 are the result of the binding of a combination of the TGA dimer and the WRKY protein as the molecular weight of such a complex (170 kD) would be close to the molecular weight of a complex of AtWRKY50-C with a TGA dimer (161 kD) as is visible in Lanes 4 and 10 (double black asterisks). The fusion product of full-length AtWRKY50 and GST (45 kD) used in these EMSAs is considerably smaller than the TGA-His dimer (76 kD), implicating that a band shift produced by the binding of a single AtWRKY50 protein to the probe would migrate to a position below the TGA dimer and intermediate of positions 1W and 2W.

Although binding of a complex of full-length AtWRKY50 with a TGA dimer is speculative, its addition to TGA2 or TGA5 resulted in an enhancement of the TGA dimer shifts (**Figure 6A**, compare the bands indicated by the single black asterisks in Lanes 6 and 12 with those in Lanes 3 and 9, respectively, and in **Figure 6C**, compare Lanes 6 and 3 in the leftmost panels). This effect was not observed with combinations of AtWRKY50-C and the TGAs (**Figure 6A**, Lanes 4 and 10; **Figure 6C**, left panels, Lanes 4). Apparently, full-length AtWRKY50 promotes binding of the TGA dimer to the 80-bp probe and to the ABC and BCD probes. This effect of AtWRKY50 on TGA binding does not require AtWRKY50’s binding site on the DNA. When combinations of full-length AtWRKY50 and TGA2 were incubated with promoter fragments lacking AtWRKY50’s binding site, the stimulating effect on TGA binding was still present. This can be seen in **Figure 6D**, where fragments B and C, containing two (LS5 and LS7) and one (LS7) CGTCA motifs, respectively, but lacking the AtWRKY50 binding site in LS4 or LS10, show an increased intensity of the TGA2 shifts in Lanes 6 of panels B and C.

## Discussion

The *PR1:LUC* based screening with 40 members of the Arabidopsis WRKY protein family showed that WRKY50 was the best activator of the *PR1* promoter. In earlier work, tobacco NtWRKY12 was shown to be involved in the expression of *PR-1a* gene (van Verk et al., 2008). Of all 74 Arabidopsis WRKY proteins, AtWRKY50 has the highest similarity to NtWRKY12, including the aberrant G-K-K sequence instead of G-Q-K immediately following the conserved W-R-K-Y sequence present in the majority of WRKY proteins. In the WRKY-DNA complex, the amino acids of the WRKY domain have been shown to be in direct contact with the DNA (Yamasaki et al., 2005). This could explain why the WK-box, NtWRKY12’s binding site in the DNA, is different from the consensus W-box. Also, AtWRKY59, one of the two other Arabidopsis WRKYs with a W-R-K-Y-G-K-K sequence was reported to lack binding specificity for the W-box (Dong et al., 2003). Here we found that AtWRKY50 binds to DNA sequences that are different from the W-box. We identified *PR1* promoter fragments A and D to specifically bind the DNA-binding domain of AtWRKY50 (**Figure 4C**). Although we haven’t performed an extensive mutational analysis to determine the minimal binding sequence, changing the two central T-residues in the TTTTC stretch in LS10 or in the GTTTC stretch in LS4 to C’s significantly reduced the binding of AtWRKY50-C, indicating that these base pairs are important for AtWRKY50’s binding. It is worth to note that NtWRKY12 and AtWRKY50, although their binding sites are different (TTTTCCAC and GACT[G]TTTC, respectively), both contain a TTTC stretch.

Our results seem to contradict an earlier finding that a C-terminal region of AtWRKY50 binds to a W-box-containing probe (Brand et al., 2010). However, in that study a mutated version of the W-box probe was also bound with significant efficiency, while the probe also contained the sequence ACTTTT, which is identical to the central part of the binding sequence we characterized in LS10. Furthermore, the authors used a 77-amino acid long C-terminal peptide, while our AtWRKY50-C consists of the C-terminal 88 amino acids. Interestingly, the corresponding 11 amino acid overlap region of NtWRKY12 is important for binding to the promoter of tobacco *PR-1a* (van Verk et al., 2011). In this regard, it is highly plausible that the extra amino acids in AtWRKY50-C contribute to the binding specificity.

Intriguingly, the inability of full-length AtWRKY50 to bind the *PR1* promoter was also observed previously with NtWRKY12 in tobacco (van Verk et al., 2008). The fact that the C-terminal His-tagged full-length AtWRKY50 protein didn’t produced a shift, it is unlikely that the binding capacity was masked by the relatively large GST-tag at the N-terminus of the protein. Also, it is plausible that the lack of correct structural folding in bacterial purified proteins show this effect. From our *in silico* modeling prediction analysis (**Figure 3D**), it is possible that the N-terminal halves of the full-length WRKYs themselves prevent binding to the DNA under EMSA conditions by stearic hindrance. The presence of bulky N-terminal region with predicted disorder (**Figure 3Dc**, white arrow) further supports our observation. We also note that work by Yamasaki et al. (2012) only studied the C-terminal region as opposed to the full-length protein. As both the tobacco and Arabidopsis homologs fail to bind full-length protein, it could indicate that this is a functionally relevant property, e.g., to prevent promiscuous binding of the WRKY protein to DNA regions with consensus binding sequences that are not in the correct structural context. It is possible that the interaction with other factors is required to change the configuration of the full-length WRKYs to release the binding domains to bind DNA. Likely candidates to bring about such a change could be TGA2 or TGA5, especially since they specifically interact with the N-terminal half of AtWRKY50 (**Figure 5B**).

Our studies in Arabidopsis protoplasts showed that AtWRKY50 enhanced expression of co-transfected *PR1::LUC* and *PR1::GUS* reporter genes and also of the endogenous *PR1* gene, suggesting that the protein acts as a transcriptional activator. This was also the case for its tobacco homolog NtWRKY12 (van Verk et al., 2008). However, while the full-length NtWRKY12-GAL4BD fusion protein activated the His reporter gene in yeast, AtWRKY50 showed no transcriptional activity in this system. We speculate that either, the BD part of the fusion protein interferes with the correct folding of AtWRKY50, or that yeast lacks specific factors necessary for its activating function. The expression of *PR1* in transgenic plants constitutively overexpressing *AtWRKY50* was increased in comparison to control plants, but only after the plants were treated with SA (**Figure 1C**). This indicates that AtWRKY50 acts as a transcriptional enhancer, but that other activators are needed to trigger the SA response of the *PR1* gene.

Previously, it was found that AtWRKY50 and AtWRKY51 are involved in repression of jasmonic acid (JA)-dependent defense responses, including *PDF1.2* marker gene expression (Gao et al., 2010). Although it was not investigated if this was the effect of a direct interaction of the WRKYs with the *PDF1.2* promoter, the authors contemplated that the WRKYs might act as transcriptional repressors, possibly by binding to specific binding sequences in the promoters of JA-responsive genes. In this context, it is significant to note that the *PDF1.2* promoter lacks W-boxes, but contains the AtWRKY50 binding element GACTGTTTC.

The C-terminal half of AtWRKY50, containing the conserved amino acid sequence WRKYGKK and a proximal zinc-finger region, bound at two positions in the 80-bp region of the *PR1* promoter essential for SA-inducible expression. This region also contains an as-1 element, consisting of two direct CGTCA motifs, that acts as a binding site for TGA transcription factors. Here we have shown that AtWRKY50 interacts with TGAs 2 and 5 in the nucleus. Using *in vitro* DNA binding assays, we showed that especially the rightmost CGTCA motif of the as-1 element was able to bind TGA2 with high efficiency and that simultaneous binding of AtWRKY50-C and TGA2 to the promoter occurred. Furthermore, although PTAs demonstrated that AtWRKY50 alone was able to activate the *PR1* promoter, and that this did not require transcription factors TGA2, TGA5, or TGA6 or their co-activator NPR1, expression of the *GUS* reporter gene was greatly enhanced when TGA2 or TGA5 were present. Our finding that TGA2 did not efficiently bind to the CGTCA motif in LS5 is at variance with the results of Després et al. (2000) who found that TGA2 binds to both LS5 and LS7. However, the authors used DNA probes containing either the LS5 or the LS7 element, which precludes a comparison of the relative strengths with which the two elements are bound. LS5 has been identified as a DNA element conferring a negative effect on *PR1* gene expression. The proximity of the TGA and AtWRKY50 binding sites in the *PR1* promoter and the ability of the proteins to interact, suggest that such an interaction could also take place when the transcription factors are bound to the promoter and that this could be relevant for *PR1* expression. Indeed, although AtWRKY50 expressed in protoplasts activates a co-transfected *PR1::GUS* gene, co-expression of TGA2 or TGA5 further enhanced GUS expression considerably. This synergistic effect was specific for TGA2 and TGA5, which both interacted with AtWRKY50, while TGA3, which did not strongly interact with AtWRKY50, was not able to enhance gene expression.

TGA2 on its own is not a transcriptional activator but requires binding of NPR1. In the absence of NPR1, TGA2 has been suggested to act as a repressor of *PR1* gene expression (Zhang et al., 2003; Rochon et al., 2006; Boyle et al., 2009). TGA2, 5 and 6 belong to the same subclass of TGAs (clade II). There is accumulating evidence that in addition to NPR1, TGAs are able to interact with other proteins. Previously, glutaredoxin was shown to interact with Arabidopsis TGA2 and tobacco TGA2.2 (Ndamukong et al., 2007), while Arabidopsis TGA2 and TGA5 were found to interact with SCL14, a protein mediating regulation of genes involved in detoxification processes (Fode et al., 2008). Based on their findings, the authors suggested that clade II TGAs could act as sequence-specific anchor proteins to recruit other transcription regulatory proteins, like SCL14 and DELLA proteins, to the promoters of their target genes. In this perspective, we speculate that TGA2 could likewise assist in recruiting AtWRKY50 to the *PR1* promoter. Also, WRKYs have been found to interact with other proteins. For example, Arabidopsis WRKY7 has been found to interact with calmodulin (CaM) through a CaM binding domain in the N-terminal half of the protein that is conserved in other members of the WRKY IId group (Park et al., 2005). Other examples are WRKY70 interacting with the EAR domain repressor ZAT7 (Ciftci-Yilmaz et al., 2007), WRKY53 interacting with mitogen activated protein kinase kinase kinase1 (MEKK1; Miao et al., 2007), WRKY33 interacting with mitogen activated protein kinase 4 (MAPK4; Andreasson et al., 2005), and WRKYs 38 and 62 that have been found to interact with histone deacetylase19 (HDA19; Kim et al., 2008). In our EMSAs, the new band shifts produced upon incubation of combinations of AtWRKY50-C and TGA2 or TGA5 with the 80-bp promoter fragment (**Figure 6A**, triple asterisks) or with probes ABC and BCD (**Figure 6C**, double asterisks) likely represent supershifts produced by the simultaneous binding of both proteins to the probe, possibly as a complex of interacting transcription factors.

In conclusion, we have shown that AtWRKY50 is an activator of Arabidopsis *PR1* expression. Its C-terminal DNA-binding domain specifically binds to two GACT(G)TTTC elements that are located at -675 and -616 bp upstream of the transcription start site in the *PR1* promoter. Complex formation between TGA2 or TGA5 and AtWRKY50 possibly functions in the regulation of *PR1* expression *in vivo*. Our study sheds new light on the intricate regulation of the key SAR gene *PR1*. The working model of the current study is summerised in Supplementary Figure S3.

## Materials and Methods

### Reporter Vector Construction

The *PR1*:: *LUC* reporter was constructed by PCR amplification of 1000bp upstream region of *PR1* (At2g14610) gene using Arabidopsis Col-0 genomic DNA as template (for primer sequences, see Supplementary Table S2). The DNA-fragments were inserted into the vector pBT10-LUC by using NcoI and EcoRI restriction enzymes. The screening was done according to Wehner et al. (2011). The *PR1::GUS* reporter was obtained by PCR on genomic DNA and cloned in front of the GUS coding region in *pT7:GUS*.

### Bacterial Expression of AtWRKY50 Fusion Proteins

The full-length and C-terminal coding sequence of AtWRKY50, AtWRKY51 and AtWRKY59 were amplified by PCR (for primer sequences, see Supplementary Table S2) and cloned in-frame with the GST open reading frame of expression vector pGEX-KG (Guan and Dixon, 1991). These plasmids were transformed into *E. coli* BL21-DE3 cells. For induction of protein expression, cultures were grown to mid-log phase at 37°C, after which isopropyl-β-thiogalactopyranoside (IPTG) was added to a final concentration of 0.1 mM and incubation continued for 3 h at 22°C. The cells were harvested by centrifugation, resuspended in 1/20th volume sonication buffer (1x phosphate-buffered saline containing 2% [v/v] Tween 20, 0.1% [v/v] Triton X-100, 5 mM dithiothreitol [DTT], and 1 mg mL-1 lysozyme) and lysed by sonication (Vibracell). The fusion proteins were purified using glutathione-Sepharose 4B columns (Amersham), which were eluted overnight at 4°C with 10 mM reduced glutathione, after which 1/50th volume Complete (Roche) protease inhibitors were added. Expressed fusion proteins were analyzed using 12% SDS-PAGE.

### Electrophoretic Mobility Shift Assay (EMSA)

Electrophoretic mobility shift assays were performed essentially as described by Green et al. (1989). DNA probes for the EMSA assays were obtained by slowly cooling down mixtures of equimolar amounts of complementary oligonucleotides from 95°C to room temperature. Annealed oligonucleotides were subsequently labeled using T4-nucleotide kinase and [γ-^32^P]ATP or using Klenow fragment and [α-^32^P]dCTP, after which unincorporated label was removed by Autoseq G-50 column chromatography (Amersham-Pharmacia Biotech). Different sets of oligonucleotides and their mutated versions are presented in Supplementary Table S2. EMSA reaction mixtures contained 0.5 μg purified protein, 3 μL 5x gel shift binding buffer [20% glycerol, 5 mM MgCl2, 2.5 mM EDTA, 2.5 mM DTT, 250 mM NaCl, 50 mM Tris-HCl, pH 7.5, 0.25 mg ml-1 poly (dI-dC) x poly (dIdC) (Promega)] in a total volume of 14 μL. After 10-min incubation at room temperature, 1 μL containing 60,000 cpm of labeled probe was added and incubation was continued for 60 min on ice. The total mixture was loaded onto a 5% polyacrylamide gel in Tris-borate buffer and electrophoresed at 4°C. After electrophoresis, the gel was dried and auto radiographed.

### qRT-PCR

Total RNA was isolated from pulverized frozen Arabidopsis tissue by phenol extraction and LiCl precipitation. Oligo (dT)-primed cDNA for PCR was obtained using M-MLV reverse transcriptase. Subsequently, qPCR was performed during 40 cycles with corresponding primers in Supplementary Table S2. The experiment was performed three times with three independent biological replicates and three technical replicates.

### Plasmid Construction and Transactivation Experiments

The *AtWRKY50* (At5g26170), *AtWRKY51* (At5g64810), *AtWRKY59* (At2g21900), *TGA2* (At5g06950) and *TGA5* (At5g06960) open reading frames were amplified by PCR using corresponding primer sets (Supplementary Table S2) from a cDNA library obtained from Arabidopsis plants 6 h after treatment with SA and cloned into pRT101. Protoplasts were prepared from Arabidopsis ecotype Columbia-0 cell suspension according to Axelos et al. (1992) with some modifications. A 5-days old cell suspension culture was diluted 5-fold in 50 mL medium (cell culture media-3.2 g/L Gamborg B5 basal medium with minimal organics [Sigma-Aldrich], 3% Sucrose, 1 μM naphthylacetic acid [NAA], pH 5.8) and incubated overnight at 25°C at 250 rpm. Cells were harvested, and cell walls digested with 20 mL of enzyme mix (0.4% macerozyme R-10, 1.5% cellulose R-10, 12% sorbitol, pH 5.8) for 3 h at 28°C with minimal shaking. The protoplasts were filtered with a 65-μm steel sieve and washed two times in 50 mL of protomedium (Gamborg B5 basal medium, 0.1 M Glc, 0.25 M mannitol, 1 μM NAA, pH 5.8). The volume of the protoplast suspension was adjusted to 4 × 10^6^ cells/mL. Protoplasts were cotransfected with 2 μg of plasmid carrying one of the *PR1 promoter::GUS* constructs and 6 μg of effector plasmid pRT101 (Töpfer et al., 1987) carrying *35S::AtWRKY50*, *35S::AtWRKY51* or *35S::AtWRKY59*. As a control, co-transformation of *PR1::GUS* fusions with the empty expression vector pRT101 was carried out. Protoplasts were transformed using polyethylene glycol as described previously (Schirawski et al., 2000). The protoplasts were harvested 16 h after transformation and frozen in liquid nitrogen. For protoplast experiments, GUS activity was determined as described (van der Fits and Memelink, 1997), with minor modifications. GUS activities from triplicate experiments were normalized against total protein level.

### Expression of TGA Fusion Proteins

The full-length coding sequence of Arabidopsis TGA2 and TGA5 were cloned in frame in front of the His-tag open reading frame of expression vector pASK-IBA45Plus (IBA Biotechnology, Göttingen, Germany). The PCR was amplified by sets of primer (Supplementary Table S2), digested with EcoRI and XhoI and cloned in pASK-IBA. These plasmids were transformed into *E. coli* BL21-(DE3) pLysS (Novagen). For induction of protein expression, cultures were grown to mid-log phase at 37°C, after which 2 mg/ml anhydrotetracyclin was added to a final concentration of 0.4 mM and incubation continued for 3 h at 29°C. The cells were harvested by centrifugation, resuspended in 20 ml binding buffer (5 mM imidazole, 0.5M NaCl, 20 mM Tris-HCl pH 8). The samples were sonicated until viscosity was low. The fusion proteins were purified using Ni-NTA agarose beads (Qiagen), which were eluted at 4°C with elution buffer (1M imidazole, 0.5M NaCl, 20 mM Tris-HCl, pH8), after which 1/50th volume Complete (Roche) protease inhibitors were added. Expressed fusion proteins were analyzed using 12% SDS-PAGE.

### *In Silico* Protein Modeling Analysis

The AtWRKY50 full length and C-terminal truncated sequences were submitted to the Phyre2 protein folding prediction server (Kelley et al., 2015) and modeled independent of any reference structure. We retrieved the final models respectively for full-length and truncated proteins as structural “pdb” file. Molecular graphics and analyses were performed with the UCSF Chimera package. Chimera is developed by the Resource for Biocomputing, Visualization, and Informatics at the University of California, San Francisco (supported by NIGMS P41-GM103311) (Pettersen et al., 2004).

### Bimolecular Fluorescence Complementation Assays

In BiFC primer sets (Supplementary Table S2) used for WRKY50 cloning with SalI and BglII in pRTL2-YNEE and –YCHA; for WRKY50 cloning with SalI and NotI in pRTL2-EEYN and –HAYC; for TGA2 cloning with Sal1 and BglII in pRTL2-YNEE and –YCHA; for TGA2 cloning with SalI and NotI in pRTL2-EEYN and –HAYC; for TGA5 cloning with Sal1 and BglII in pRTL2-YNEE and –YCHA; for TGA5 cloning with SalI and NotI in pRTL2-EEYN and –HAYC. PCR-amplified inserts were digested with the restriction enzymes mention above and cloned in the respective pRTL2 derivates (Bracha-Drori et al., 2004) digested with the corresponding enzymes. Protoplasts were isolated and transformed with PEG as described above. Images of transfected protoplasts were acquired with a Leica DM IRBE confocal laser-scanning microscope equipped with an Argon laser line of 488 nm (excitation) and a band pass emission filter of 500–550 nm.

### Yeast Two Hybrid Assays

Full-length AtWRKY50 (At5g26170) and AtNPR1 (At1g64280) cloned in pAS2.1 with primer sets (Supplementary Table S2) digested with EcoRI and BamHI, cloned into pAS2.1 and were co-transformed to yeast strain PJ69-4A (James et al., 1996). The TGA2 (At5g06950) and the TGA5 (At5g06960) ORF was PCR-amplified from Arabidopsis cDNA library using the primer sets (Supplementary Table S2), digested with EcoRI and BamHI and cloned into pACT2. For auto activation assays, transformants were plated on minimal synthetic defined (SD)-glucose medium supplemented with Met/Ura/His and lacking Leu and Trp (-LT). Ability to activate transcription in yeast was evaluated by monitoring growth after 7 days on selective SD medium lacking Leu, Trp and His (-LTH). Interaction assays were performed by co-transformation of bait and prey plasmids into yeast strain PJ69-4A and plated on SD-LT medium. As control, empty pAS2.1 and pACT2 were used. Transformants were allowed to grow for 4–5 days. Subsequently, cells were incubated for 16 h in liquid SD-LT and 10 μL of 10-fold dilutions were spotted on SD-LTH medium. Yeast cells were allowed to grow for 7 days at 30°C.

## Data Availability

The datasets and scripts generated during the current study are available from the corresponding author on request.

## Author Contributions

RH designed and performed the experiments. RH, AS, IH, and HL wrote the manuscript. MQ helped with *in silico* modeling analysis. HL supervised this study.

## Conflict of Interest Statement

The authors declare that the research was conducted in the absence of any commercial or financial relationships that could be construed as a potential conflict of interest.
