# Supplementary material for: Arabidopsis WRKY50 and TGA Transcription Factors Synergistically Activate Expression of PR1
Source: Front Plant Sci. 2018 Jul 13;9:930. doi: 10.3389/fpls.2018.00930 (PMC6053526; doi:10.3389/fpls.2018.00930)
Supplement: Supplementary file 1 [file Data_Sheet_1.docx]

**Arabidopsis WRKY50 and TGA transcription factors synergistically activate expression of *PR1***

Rana M.F. Hussain^1^ , Arsheed H. Sheikh^2*^, Imran Haider^1^, Mussa Quareshy^2^, Huub J.M. Linthorst^1^

**Supplementary Fig. S1.** Western blot with GST-tagged C-terminal and full-length AtWRKY50, -51 and -59, as indicated above the lanes. Bands corresponding to the respective longest peptides are indicated with single (C-termini) or double (full-length) asterisks and with C-term* and FL** to the left of the panel. The size (kDa) of molecular weight markers is indicated to the right of the panel.


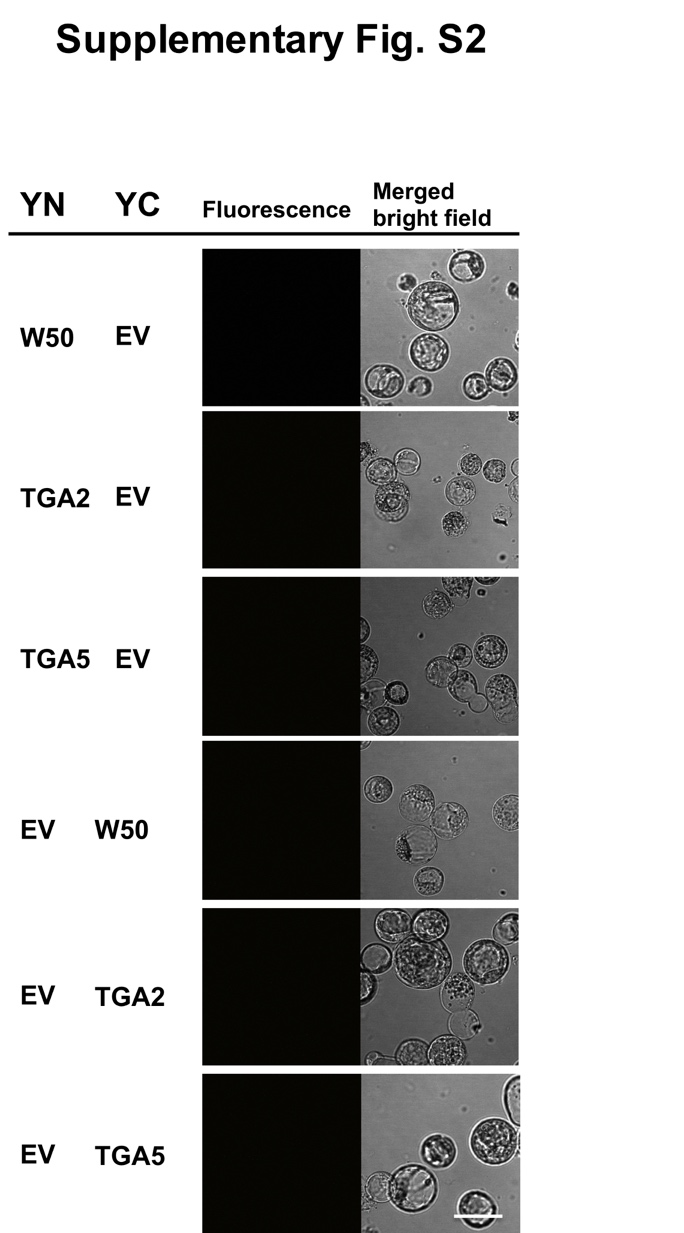


**Supplementary Fig. S2.** BiFC analysis of N-terminal and C-terminal of the indicated proteins with empty vectors. The lack of fluorescent signal served as negative control for the experiments performed in Fig. 5C (Scale bar = 10µm).

**Supplementary Fig. S3.** Propsed working model of WRKY50 and TGA activation of *PR1*.

**TABLES**

**Supplementary Table 1. Protoplast transactivation assays**

| Name | Gene | Fold induction |
| --- | --- | --- |
| AtWRKY50 | At5g26170 | 6.03 |
| AtWRKY42 | At4g04450 | 5.74 |
| AtWRKY26 | At5g07100 | 2.51 |
| AtWRKY28 | At4g18170 | 2.38 |
| AtWRKY10 | At1g55600 | 2.33 |
| AtWRKY35 | At2g34830 | 2.23 |
| AtWRKY25 | At2g30250 | 2.07 |
| AtWRKY47 | At4g01720 | 2.02 |
| AtWRKY6 | At1g62300 | 1.98 |
| AtWRKY17 | At2g24570 | 1.78 |
| AtWRKY38 | At5g22570 | 1.74 |
| AtWRKY22 | At4g01250 | 1.52 |
| AtWRKY44 | At2g37260 | 1.49 |
| AtWRKY12 | At2g44745 | 1.49 |
| AtWRKY46 | At2g46400 | 1.49 |
| AtWRKY75 | At5g13080 | 1.48 |
| AtWRKY43 | At2g46130 | 1.44 |
| AtWRKY72 | At5g15130 | 1.42 |
| AtWRKY21 | At2g30590 | 1.42 |
| AtWRKY29 | At4g23550 | 1.38 |
| AtWRKY45 | At3g01970 | 1.37 |
| AtWRKY33 | At2g38470 | 1.36 |
| AtWRKY55 | At2g40740 | 1.36 |
| AtWRKY41 | At4g11070 | 1.34 |
| AtWRKY9 | At1g68150 | 1.34 |
| AtWRKY69 | At3g58710 | 1.28 |
| AtWRKY70 | At3g56400 | 1.26 |
| AtWRKY1 | At2g04880 | 1.23 |
| AtWRKY15 | At2g23320 | 1.19 |
| AtWRKY61 | At1g18860 | 1.13 |
| AtWRKY20 | At4g26640 | 1.13 |
| AtWRKY56 | At1g64000 | 1.12 |
| AtWRKY23 | At2g47260 | 1.11 |
| AtWRKY13 | At4g39410 | 1.10 |
| AtWRKY67 | At1g66550 | 1.08 |
| AtWRKY65 | At1g29280 | 1.05 |
| AtWRKY40 | At1g80840 | 1.03 |
| AtWRKY7 | At4g24240 | 0.97 |
| AtWRKY62 | At5g01900 | 0.97 |
| AtWRKY53 | At4g23810 | 0.90 |
| AtWRKY60 | At2g25000 | 0.84 |

**Supplementary Table 2. List of primers used in this study**

| **For Reporter assay** | | |
| --- | --- | --- |
| PR1::LUC | F | GTGGAATTCCTGATTCGGAGGGAGTATATGTTATTG |
|  | R | CGATCCATGGTTTTCTAAGTTGATAATGGTTATTG |
| PR1::GUS | F | GTCAAAGCTTCTGATTCGGAGGGGTATATGTTATTG |
|  | R | CGATGGATCCTTTTCTAAGTTGATAATGGTTATTGTTGTG |
| AtWRKY50 pRT101 | F | ATAGCTCGAGGTATGAATGATGCAGACACAAACTTG |
|  | R | GCCTCTAGACGAGTCTTAGTTCATGCTTGAGTGATTGTG |
| AtWRKY51 pRT101 | F | AAACGAATTCAAATGAATATCTCTCAAAACCCTAGCC |
|  | R | GATGAGGTACCTGGATTAAGATCGAAGAAGGTGTTG |
| AtWRKY59 pRT101 | F | GAGACTCGAGAAATGAACTATCCTTCAAACCCTAACC |
|  | R | CTACTCTAGATCATTATGGAGCAGAATGAGAGAGAAAC |
| TGA2- pRT101 | F | TAGCGAATTCGATGGCTGATACCAGTCCGAG |
|  | R | TGACGGATCCGGTCACTCTCTGGGTCGAGCAAGC |
| TGA3- pRT101 | F | TAGCGAATTCGATGGAGATGATGAGCTCTTC |
|  | R | TGACGGATCCGGTCAAGTGTGTTCTCGTGGACGTG |
| TGA5- pRT101 | F | TAGCGAATTCGATGGGAGATACTAGTCCAAG |
|  | R | TGACGGATCCGGTCACTCTCTTGGTCTGGCAAGC |
| **For qRT-PCR** | | |
| *PR1* | F | GTTCTTCCCTCGAAAGCTCAAGAT |
|  | R | CACCTCACTTTGGCACATCCG |
| tubulin7 | F | GGAAGAAGCTGAGTACGAGCA |
|  | R | GCAACTGGAAGTTGAGGTGTT |
| actin3 | F | CCTCATGCCATCCTCCGTCT |
|  | R | CAGCGATACCTGAGAACATAGTGG |
| **For cloning WRKYs** | | |
| AtWRKY50-BD::GST | F | CTAGAATTCCTGCCGACAACCAAAACAAG |
|  | R | GCCAAGCTTCGAGTCTTAGTTCATGCTTGAGTGATTGTG |
| AtWRKY50-FL::GST | F | ATAGGAATTCGTATGAATGATGCAGACACAAACTTG |
|  | R | GCCAAGCTTCGAGTCTTAGTTCATGCTTGAGTGATTGTG |
| AtWRKY51-BD::GST | F | CTAGAATTCGAGGAAGTAAAGAGAGTGATCAG |
|  | R | GATGAAAGCTTTGGATTAAGATCGAAGAAGAGAGTGTTGG |
| AtWRKY51-FL::GST | F | AAACGAATTCAAATGAATATCTCTCAAAACCCTAGC |
|  | R | GATGAAAGCTTTGGATTAAGATCGAAGAAGAGAGTGTTGG |
| AtWRKY59-BD::GST | F | CTAGAATTCGGAAGAGACACAAAGAAGATCCG |
|  | R | CTACAAGCTTTCAATATGGAGCAGAATGAGAGAAAC |
| AtWRKY59-FL::GST | F | GAGAGAATTCAAATGAACTATCCTTCAAACCCTAACC |
|  | R | CTACAAGCTTTCAATATGGAGCAGAATGAGAGAAAC |
| **For EMSA** | | |
| PR1 80BP | F | ATCGGATCCGGTGATCTATTGACTGTTTCTCTAC |
|  | R | GCCTAGATCTGAAAAGTCCTGAAGAATATATGCC |
| PR1 fragA | F | GGTGATCTATTGACTGTTTCTCTACGTCACTA |
|  | R | TAGTGACGTAGAGAAACAGTCAATAGATCACC |
| PR1 fragB | F | TTTCTCTACGTCACTATTTTACTTACGTCATA |
|  | R | TATGACGTAAGTAAAATAGTGACGTAGAGAAA |
| PR1 fragC | F | TTTTACTTACGTCATAGATGTGGCGGCATATA |
|  | R | TATATGCCGCCACATCTATGACGTAAGTAAAA |
| PR1 fragD | F | GATGTGGCGGCATATATTCTTCAGGACTTTTC |
|  | R | GAAAAGTCCTGAAGAATATATGCCGCCACATC |
| Frag Am1 | F | GGGGGTGATCTATTGACTTTTCTCTACGTCACTAT |
|  | R | GGGATAGTGACGTAGAGAAAAGTCAATAGATCACC |
| Frag Am2 | F | GGGGGTGATCTATTGACTGCCTCTCTACGTCACTA |
|  | R | GGGTAGTGACGTAGAGAGGCAGTCAATAGATCACC |
| Frag Dm1 | F | GGGGATGTGGCGGCATATATTCCCCAGGACTTTTC |
|  | R | GGGGAAAAGTCCTGGGGAATATATGCCGCCACATC |
| Frag Dm2 | F | GGGGATGTGGCGGCATATATTCTTCAGGACCCTTC |
|  | R | GGGGAAGGGTCCTGAAGAATATATGCCGCCACATC |
| PR-2 LS10 | F | GGGCATATTGTTAGACTTTTCAAAGCGTATATT |
|  | R | GGGAATATACGCTTTGAAAAGTCTAACAATATG |
| Frag. ABC | F | ATCGGATCCGGTGATCTATTGACTGTTTCTCTAC |
|  | R | TATATGCCGCCACATCTATGACGTAAGTAAAA |
| Frag. BCD | F | TTTCTCTACGTCACTATTTTACTTACGTCATA |
|  | R | GCCTAGATCTGAAAAGTCCTGAAGAATATATGCC |
| Frag. ABC LS5m | F | ATCGGATCCGGTGATCTATTGACTGTTTCTCTAC |
|  | R | CATCTATGACGTAAGTAAAATAGTTGCGTAGAG |
| Frag. ABC LS7m | F | ATCGGATCCGGTGATCTATTGACTGTTTCTCTAC |
|  | R | CATCTATTGCGTAAGTAAAATAGTGACGTAGAG |
| Frag. BCD LS5m | F | CTCTACGCAACTATTTTACTTACGTCATAGATG |
|  | R | GCCTAGATCTGAAAAGTCCTGAAGAATATATGCC |
| Frag. BCD LS7m | F | CTCTACGTCACTATTTTACTTACGCAATAGATG |
|  | R | GCCTAGATCTGAAAAGTCCTGAAGAATATATGCC |
| Frag. ABC LS5+7m | F | ATCGGATCCGGTGATCTATTGACTGTTTCTCTAC |
|  | R | CATCTATTGCGTAAGTAAAATAGTTGCGTAGAG |
| Frag. BCD LS5+7m | F | CTCTACGCAACTATTTTACTTACGCAATAGATG |
|  | R | GCCTAGATCTGAAAAGTCCTGAAGAATATATGCC |
| **For cloning TGAs** | | |
| TGA2 | F | TAGCGAATTCGATGGCTGATACCAGTCCGAG |
|  | R | TGACCTCGAGGGCTCTCTGGGTCGAGCAAGC |
| TGA5 | F | TAGCGAATTCGATGGGAGATACTAGTCCAAG |
|  | R | TGACCTCGAGGGCTCTCTTGGTCTGGCAAGC |
| **For BiFC Assay** | | |
| W50-pRTL2  YNEE&YCHA | F | GATCGTCGACAATGAATGATGCAGACACAAACTTG |
|  | R | CAGTAGATCTGTTAGTTCATGCTTGAGTGATTGTG |
| W50-pRTL2  EEYN&HAYC | F | GATCGTCGACAATGAATGATGCAGACACAAACTTG |
|  | R | CGTAAGCGGCCGCGTGTTCATGCTTGAGTGATTGT |
| TGA2-pRTL2  YNEE&YCHA | F | GATCGTCGACAATGGCTGATACCAGTCCGAGAACT |
|  | R | CAGTAGATCTGTCACTCTCTGGGTCGAGCAAGCCA |
| TGA2-pRTL2  EEYN&HAYC | F | GATCGTCGACAATGGCTGATACCAGTCCGAGAACT |
|  | R | CGTAAGCGGCCGCGTCTCTCTGGGTCGAGCAAGCC |
| TGA5-pRTL2  YNEE&YCHA | F | GATCGTCGACAATGGGAGATACTAGTCCAAGAACA |
|  | R | GATCGTCGACAATGGGAGATACTAGTCCAAGAACA |
| TGA5-pRTL2  EEYN&HAYC | F | GATCGTCGACAATGGGAGATACTAGTCCAAGAACA |
|  | R | CGTAAGCGGCCGCGTCTCTCTTGGTCTGGCAAGCC |
| **For Yeast two hybrid assays** | | |
| W50-pAS2.1 | F | ATAGGAATTCGTATGAATGATGCAGACACAAACTTG |
|  | R | GCCGGATCCCGAGTCTTAGTTCATGCTTGAGTGATTGTG |
| AtNPR1-pAS2.1 | F | TAGCGAATTCTAATGGACACCACCATTGATGG |
|  | R | TGACGGATCCTCACCGACGACGATGAGAG |
| TGA2-pACT2 | F | TAGCGAATTCGATGGCTGATACCAGTCCGAG |
|  | R | TGACGGATCCGGTCACTCTCTGGGTCGAGCAAGC |
| TGA5-pACT2 | F | TAGCGAATTCGATGGGAGATACTAGTCCAAG |
|  | R | TGACGGATCCGGTCACTCTCTTGGTCTGGCAAGC |
